# A Look inside the Replication Dynamics of SARS-CoV-2 in Blyth’s Horseshoe Bat (*Rhinolophus lepidus*) Kidney Cells

**DOI:** 10.1128/spectrum.00449-22

**Published:** 2022-05-31

**Authors:** Heidi Auerswald, Dolyce H. W. Low, Jurre Y. Siegers, Teyputita Ou, Sonita Kol, Saraden In, Martin Linster, Yvonne C. F. Su, Ian H. Mendenhall, Veasna Duong, Gavin J. D. Smith, Erik A. Karlsson

**Affiliations:** a Virology Unit, Institut Pasteur du Cambodge, Institut Pasteur International Network, Phnom Penh, Cambodia; b Programme in Emerging Infectious Diseases, Duke-NUS Medical School, Singapore; c SingHealth Duke-NUS Global Health Institute, SingHealth Duke-NUS Academic Medical Centre, Singapore; d Duke Global Health Institute, Duke University, Durham, North Carolina, USA; University of Georgia

**Keywords:** SARS-CoV, bat cells, reservoir, coronavirus, bat, primary cell, SARS-CoV-2, viral replication, virus isolation, zoonoses

## Abstract

Bats are considered the natural reservoir of numerous emerging viruses such as severe acute respiratory syndrome coronaviruses (SARS-CoVs). There is a need for immortalized bat cell lines to culture and investigate the pathogenicity, replication kinetics, and evolution of emerging coronaviruses. We illustrate the susceptibility and permissiveness of a spontaneously immortalized kidney cell line (Rhileki) from Blyth’s horseshoe bat (*R. lepidus*) to SARS-CoV-2 virus, including clinical isolates, suggesting a possible virus-host relationship. We were able to observe limited SARS-CoV-2 replication in Rhileki cells compared with simian VeroE6 cells. Slower viral replication in Rhileki cells was indicated by higher ct values (RT-PCR) at later time points of the viral culture and smaller foci (foci forming assay) compared with those of VeroE6 cells. With this study we demonstrate that SARS-CoV-2 replication is not restricted to *R. sinicus* and could include more *Rhinolophus* species. The establishment of a continuous *Rhinolophus lepidus* kidney cell line allows further characterization of SARS-CoV-2 replication in *Rhinolophus* bat cells, as well as isolation attempts of other bat-borne viruses.

**IMPORTANCE** The current COVID-19 pandemic demonstrates the significance of bats as reservoirs for severe viral diseases. However, as bats are difficult to establish as animal models, bat cell lines can be an important proxy for the investigation of bat-virus interactions and the isolation of bat-borne viruses. This study demonstrates the susceptibility and permissiveness of a continuous kidney bat cell line to SARS-CoV-2. This does not implicate the bat species *Rhinolophus lepidus*, where these cells originate from, as a potential reservoir, but emphasizes the usefulness of this cell line for further characterization of SARS-CoV-2. This can lead to a better understanding of emerging viruses that could cause significant disease in humans and domestic animals.

## INTRODUCTION

Bats are considered the natural reservoir of numerous emerging viruses like severe acute respiratory syndrome coronaviruses (SARS-CoVs) (1, 2), including SARS-CoV-2 that is responsible for the ongoing Coronavirus Disease 2019 (COVID-19) pandemic (3–6). To date, the SARS-related CoV (SARSr-CoV) RaTG13 virus, isolated from feces of Rhinolophus affinis bats in Yunnan province, China has the highest sequence identity (96.1%) to SARS-CoV-2 (3, 7). Several other viruses belonging to the RaTG13/SARS-CoV-2 lineage have been recently identified in other *Rhinolophus* bat species in China (*R. malayanus* [7], *R. pusillus* [5]), Cambodia (*R. shameli* [6]), Thailand (*R. acuminatus* [4]), and Laos (*R. malayanus*, *R. pusillus*, and *R. marshalli* [8]) with a sequence identity ranging from 91.5% to 96.8%. Together, the observed confined host range suggests the presence of a diversity of SARS-CoV-2 related viruses circulating in *Rhinolophus* species in Southeast Asia.

There is a need for immortalized bat cell lines to culture, and investigate the pathogenicity, replication kinetics, and evolution of emerging coronaviruses. Only few *in vitro* models support replication of SARS-CoV-2 in *Rhinolophus* cells and including *R. sinicus* lung and brain cells, and cells of other bat species such as Pipistrellus abramus kidney cells. Overall, viral titers remained rather low compared with nonbat cell lines like VeroE6 (9) (Table 1; Table S1). So far, the only efficient *in vitro* system for the replication of SARS-CoV-2 in bats cells are intestinal organoids generated from *R. sinicus* (10) further confirming the permissiveness of *Rhinolophus* bats to SARS-CoV-2 and related viruses. However, the generation of primary bat organoid systems remains laborious and time-consuming. In addition, intestinal organoid systems can display a high heterogeneity with different expression levels of ACE2 leading to variable susceptibilities to SARS-CoV-2 infection as seen in human intestinal organoids from different donors (11).

**TABLE 1:** Summary of reports on SARS-CoV-2 culture in *Rhinolophus* cells

Species	Organ	SARS-CoV-2 strain	MOI	CPE	Growth	Assay	Reference
Chinese rufous horseshoe bat *(R. sinicus)*	Lung	HK20	0.1	none	1.08 log_10_	qRT-PCR	9
	Brain	HK20	0.1	none	1.46 log_10_	qRT-PCR	9
	Kidney	HK20	0.1	none	0.28 log_10_	qRT-PCR	9
	Lung	HKU-001a	0.1	none	none	qRT-PCR	17
	Kidney	HKU-001a	0.1	none	none	qRT-PCR	17
	Intestinal organoid	N/D	0.1	yes	>3 log_10_; >6 log_10_	qRT-PCR; TCID50	10
Greater horseshoe bat *(R. ferrum-equinum)*	Skin (patagium)	BetaCoV/France/ IDF0372/2020	1	n/d^*a*^	none	FACS	37

a n/d, not determined.

Blyth’s horseshoe bat (*Rhinolophus lepidus*) is a species of the *Rhinolophidae* family widely distributed across South and Southeast Asia. So far, these bats are not associated with coronaviruses or SARSr-CoVs (12, 13) although it is likely that coronaviruses are present in this species based on abundance of CoVs found in other *Rhinolophus* species, e.g., *R. pusillus* and *R. monoceros* (14). Here, we describe infection and sustained virus replication of SARS-CoV-2 from clinical isolates in a newly established *Rhinolophus lepidus* kidney (Rhileki) cell line.

## RESULTS

### Replication of SARS-CoV-2 virus in Rhileki cells.

**FIG 1:**
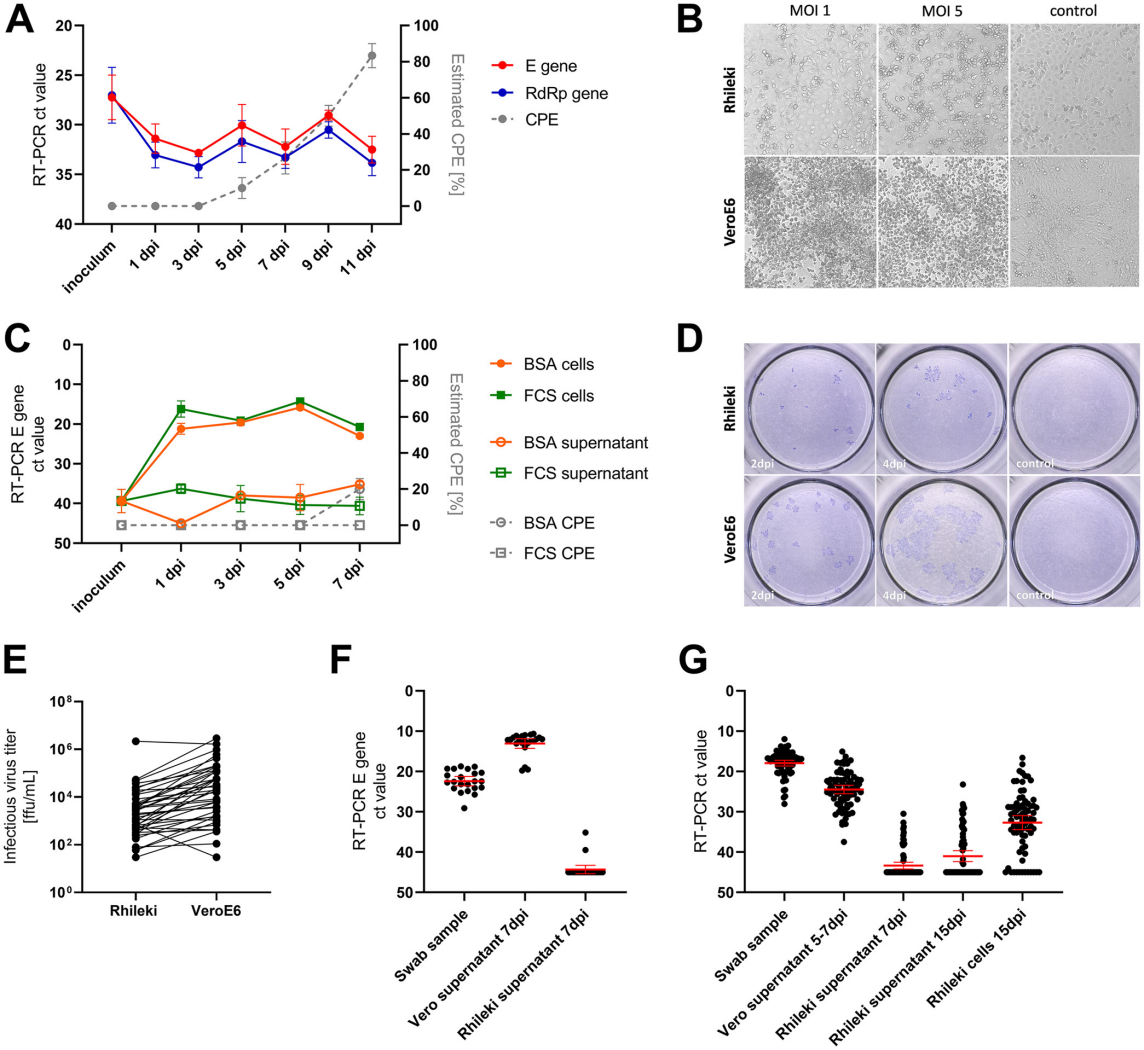
SARS-CoV-2 infection of Rhileki cells. (A) SARS-CoV-2 quantified Rhileki cell supernatants. Mean ct values with SEM of Rhileki cell culture supernatants infected with SARS-CoV-2 MOI 1 (merged results of isolates 1775, 2018, and 2310) previously passaged six times in Vero cells. Replication was determined by E gene (red) and RdRp expression (blue). Cytopathic effect (CPE) of cells was estimated through observation by bright field microscopy at ×10 magnification (gray dotted lines). (B) Cytopathic effect upon SARS-CoV-2 infection of Rhileki and VeroE6 cells 4 dpi. Cell monolayers were inoculated with the Cambodian SARS-CoV-2 isolated 1775 at MOI of 1 or 5 for 1 h; afterwards, cells were washed and grown in infection medium containing 5% FCS (for Vero cells) or 0.5% BSA (for Rhileki cells), respectively. CPE was documented by bright field microscopy at a ×10 magnification. Images were taken with Cytation5 multi-mode reader (BioTek). (C) Rhileki cells were inoculated with SARS-CoV-2 MOI 0.1 (merged results of isolates 1775, 2018, and 2310) using different media compositions either with 0.5% BSA (orange circles) or with 5% FCS (green squares). Replication was determined by E gene expression in the culture supernatants (unfilled shapes) and cell lysates (filled shapes). Cytopathic effect (CPE) of cells was estimated through observation by bright field microscopy at ×10 magnification (gray dotted lines). (D) Focus forming assay on Rhileki and VeroE6 cells infected with SARS-CoV-2 MOI 0.1 (isolate 2310, passaged six times in Vero cells). Staining of infected cells was carried out with pooled sera from confirmed COVID-19 patients. (E) Rhileki and VeroE6 cells were infected with the same amount of 47 Wuhan-like virus stock samples. Virus titers were determined by FFA staining, expressing the amount of initial infection events in focus forming units (ffu/mL). Wilcoxon test *P* < 0.0001. (F) Isolation attempts of Wuhan strains. Combined oro-nasopharyngeal swab samples of confirmed COVID-19 patients were used for inoculation and culture supernatants of both cell lines were analyzed 7 dpi for the presence of SARS-CoV-2 by E gene RT-PCR. (G) Isolation of α-VoC strains. SARS-CoV-2 in swabs used for inoculation was determined by IP4 RT-PCR. Supernatant of isolation on Vero cells were taken as soon as 50% CPE was observed (5 to 7 dpi). For isolation on Rhileki cells, supernatant was taken and analyzed from 3 dpi on every 2 days. Additionally at 15 dpi supernatant and lyzed cells were tested for presence of SARS-CoV-2 by RT-PCR targeting E gene.

A multistep growth curve showed SARS-CoV-2 replication in Rhileki cells for three Cambodian SARS-CoV-2 isolates (Wuhan strains) that had first been passaged six times in Vero cells (Fig. 1A). Overall, the viral load in the culture supernatant increased slightly from 1 dpi (E gene mean ct: 31.39; 95% CI = 25.04 to 37.75) to 9 dpi (E gene mean ct: 29.03; 95% CI = 26.89 to 31.18), with the monitoring of the RdRp gene showing a similar trend. After 9 dpi, the viral loads in the culture supernatant dropped, coinciding with increasing cell death. Infection of Rhileki cells with SARS-CoV-2 at multiplicity of infection (MOI) 1 and 5 resulted in the detachment of the cell monolayer, indicative of cytopathic effect (CPE), a hallmark of SARS-CoV-2 infection (Fig. 1B). To determine optimal growth conditions for SARS-CoV-2 in Rhileki cells, we performed comparative viral growth curves with the 0.5% bovine serum albumin (BSA)-containing medium and the 5% fetal calf serum (FCS) medium (Fig. 1C). As the viral load in the supernatant of infected Rhileki cells was low, we also determined the viral load within the cells. Under both conditions, the viral load is higher within the cells than in the supernatant. The amount of released SARS-CoV-2 that can be found in the supernatant increased when grown with 0.5% BSA but decreased after 1 dpi when maintained with 5% FCS. Focus forming assays (FFA) confirmed infection of Rhileki cells, but the size of foci formed by SARS-CoV-2-infected Rhileki cells were smaller than those formed on infected VeroE6 cells (Fig. 1D) indicating a slower viral replication rate in Rhileki cells. To investigate that further, Rhileki and VeroE6 cells were simultaneously infected with the same amount of 47 virus stock samples (p1 from Vero culture; infection of 2*10^4^ cells with 50 μL virus stock) of Wuhan-like viruses and the infection rate was measured by FFA (Fig. 1E). The infection rate (measured 4 dpi), expressing the amount of initial infection events, was found to be lower in Rhileki cells (mean 5.22*10^4^ ffu/mL) than in VeroE6 cells (mean 1.73*10^5^).

### Isolation of SARS-CoV-2 in Rhileki cells.

Next, we investigated whether Rhileki cells are suitable for isolation of SARS-CoV-2 virus from clinical samples. Clinical samples that tested positive for SARS-CoV-2 RNA by qRT-PCR and previously used for successful virus isolation on Vero cells were used for isolation in Rhileki cells. A total of 22 “Wuhan” swab samples, collected in Q2 of 2020 (ct range 18.8 to 29.1, mean 22.39), and 75 α-VoC swab samples from Q2 of 2021 (ct range: 12.0 to 28.1, mean: 17.94) were used (Table 2). None of the isolation attempts in Rhileki cells with the Wuhan clade clinical specimens were successful (Fig. 1F; Table S2). This unsuccessful virus isolation on Rhileki cells might be due to insufficient sample quality as the isolation on Vero cells was performed with clinical samples frozen only once and stored for no longer than 3 months, whereas the isolation on Rhileki cells was performed later with the same clinical samples (refrozen twice, stored up to 12 months). Therefore, another attempt with unfrozen clinical α-VoC swab samples was performed with simultaneous inoculation of Vero and Rhileki cells. Additionally, based on the previous findings of a slower viral replication rate in Rhileki cells, the isolation experiments in these cells were prolonged from 7 dpi to 15 dpi. Of the α-VoC swab samples, 26 (34.7%) were able to infect and replicate in Rhileki cells defined as positive qRT-PCR at 15 dpi in both the supernatant and cells (Fig. 1G; Table S3). Like the previously described viral culture experiments with established SARS-CoV-2 isolates (Fig. 1C), the viral load was higher within the Rhileki cells than in the virus culture supernatant.

**TABLE 2:** SARS-CoV-2 isolation rate in Rhileki and Vero cells

Variant		Isolation in Vero cells	Isolation in Rhileki cells	
E gene mean ct value (95% CI)^*a*^	Oro-nasopharyngeal swab sample	Culture supernatant	Culture supernatant	Cells
Wuhan SARS-CoV-2 (*n* = 22)	22.39 (21.22 to 23.56)	13.06^*c*^ (11.85 to 14.26)	44.30^*d*^ (43.25 to 45.35)	n/d^*e*^
α-VoC SARS-CoV-2 (*n* = 75)	17.94^*b*^ (17.24 to 18.65)	24.49^*e*^ (23.46 to 25.51)	41.01^*f*^ (39.66 to 42.36)	32.69^*c*^ (30.94 to 34.43)

a PCR threshold was set at ct = 45.

b IP4.

c 6 to 7 dpi.

d 7 dpi.

e n/d, not determined.

f 15 dpi.

## DISCUSSION

The development of novel bat cell lines is pivotal for the understanding of virus-bat interactions because bats function as a reservoir species for many emerging zoonotic viruses, especially coronaviruses (15). Bats of the genus *Rhinolophus* are considered a reservoir species for many different coronaviruses, including SARS-CoV-related viruses (4–7, 14). We were able to observe limited SARS-CoV-2 replication in a kidney cell line originated from *R. lepidus*. So far, this species was not identified as a reservoir for coronaviruses. However various alpha- and beta-coronaviruses were found in other closely related, small horseshoe bats of the species *R. pusillus* and *R. monocerus* in China (14, 16).

While SARS-CoV was shown to successfully replicate in kidney cells from *R. sinicus*, SARS-CoV-2 failed to replicate in these (9, 17) (Table 1). Only slight increases in virus replication in *R. sinicus* lung (1.08 log_10_-fold) and *R. sinicus* brain cells (1.46 log_10_-fold) were documented before (9). Although viral replication observed during our investigation was not as efficient as in Vero cells and in *R. sinicus* bat intestinal organoids (10), we demonstrated that SARS-CoV-2 replication is not restricted to *R. sinicus* and could include more *Rhinolophus* species. Slower viral replication in Rhileki cells was indicated by higher ct values (RT-PCR) at later time points of the viral culture and smaller foci (FFA) compared with the results of Vero/VeroE6 cells. This could be the result of higher basal levels of interferon and the expression of interferon stimulated genes, a feature frequently observed in bat cells (18–20), in contrast to the type I interferon-deficient Vero/VeroE6 cells (21–23). The limited replication could also be a result of a lower initial infection rate caused by a lower affinity of the *R. lepidus* ACE2 to the receptor-binding domain (RBD) of SARS-CoV-2, similar to what was observed for the ACE2 of *R. macrotis* (24). The successful infection of Rhileki cells with multiple SARS-CoV-2 clinical isolates suggests the presence of functional bat ACE2 and TMPRSS2 in these cells, which is necessary for virus attachment and fusion (25, 26). Additionally, the isolation and passaging of SARS-CoV-2 can lead to adaptive mutations, as seen for SARS-CoV-2 passaged in Vero cells that resulted in deletions in the viral multibasic cleavage site (27, 28). Therefore, this study not only used established (passaged) isolates but also original patient samples to proof the susceptibility of Rhileki cells to SARS-CoV-2. An investigation of the effect of human coronavirus (hCoV) 229E grown in Rhileki cells also revealed that virus isolates acquired deletions in the spike and ORF4 genes upon serial passaging (29).

Similar to SARS-CoV and MERS-CoV, the susceptibility and permissiveness of bat cells to SARS-CoV-2 varies between species but also between virus strains/clades (Table 1; Table S1) (30). Lau et al. demonstrated significant SARS-CoV-2 replication in lung, brain, and kidney cells of *R. sinicus* and in kidney cells of Pipistrellus abramus, and to a much lesser extent (<1 log_10_ increase in viral titer in culture supernatant determine by qRT-PCR 5 dpi) in kidney cells of Miniopterus pusillus, Tylonycteris pachypus, and *Myotis ticketii*, as well as in intestine, brain, and kidney cells of Rousettus leschenaultii (9). The same study also failed to observe replication of hCoV 229E in their bat cells, whereas Rheliki cells were able to productively produce 229E (29). Other studies observed no SARS-CoV-2 replication for cells of *Molossidae*, *Pteropodidae*, and *Vespertilionidae* bats (Table S1). Besides bats of the *Rhinolophus* family, SARSr-CoVs were also reported in *Hipposideridae* bats of the species *Aselliscus stoliczkanus* (14, 31) and Hipposideros armiger (14, 32) from China. Therefore, establishment of continuous bat cell lines, including those from non-*Rhinolophus* bat species, are important for studying viruses of pandemic concern in their natural hosts.

Understanding bat-virus interactions and the isolation of bat-borne viruses are important for understanding emerging viruses that could cause significant disease in humans and domestic animals (15). Here, we describe the susceptibility and permissiveness of kidney cells from Blyth’s horseshoe bat (*R. lepidus*) to SARS-CoV-2, including clinical isolates, suggesting a possible virus-host relationship. This work demonstrates the usefulness of the Rhileki cell line for further characterization of the SARS-CoV-2 replication in *Rhinolophus* bat cells, as well as for isolation attempts of other bat borne viruses.

## MATERIALS AND METHODS

### Establishment of Rhileki cell line.

A spontaneously immortalized and clonal cell line was generated from kidney tissue of a *Rhinolophus lepidus* bat (NUS-IACUC B01/12) as described before (29) and named Rhileki for *Rhinolophus lepidus* kidney.

### Cells and viruses.

VeroE6 (ATCC CRL-1586) and Vero (ATCC CCL-81) cells were maintained in Dulbecco’s modified Eagle medium (DMEM; Sigma-Aldrich) supplemented with 10% FCS (Gibco, Gaithersburg, MD, USA), and 100 U/mL penicillin-streptomycin (Pen/Strep; Gibco) at 37°C and 5% CO_2_. Rhileki cells were maintained in DMEM supplemented with 10% FCS, 1% nonessential amino acids (Gibco), 1 mM sodium pyruvate (Gibco), and 100 U/mL Pen/Strep at 37°C and 5% CO_2_.

SARS-CoV-2 isolation and culture was performed using Vero cells with DMEM containing 5% FCS, and 100 U/mL Pen/Strep, or using Rhileki cells with DMEM containing 0.5% bovine serum albumin (BSA; Gibco), 1% nonessential amino acids (Gibco), 1 mM sodium pyruvate (Gibco), and 100 U/mL Pen/Strep. Isolation attempts were performed by incubating cells for up to 7 days (for Vero cells), or up to 15 days (for Rhileki cells) with filtrated (0.45 μm) combined nasopharyngeal/oropharyngeal swab samples from individual patients. For subsequent virus culture experiments of successfully isolated strains, inoculation was restricted to 1 h at 37°C and 5% CO_2_, followed by a washing step of the cells with DMEM. Established Cambodian ancestral Wuhan SARS-CoV-2 isolates 1775 (GISAID: EPI_ISL_956384), 2018 (GISAID: EPI_ISL_956389), and 2310 (GISAID: EPI_ISL_956394) passaged up to six times in Vero cells were used for comparative cultivation experiments.

### SARS-CoV-2 patient samples.

Combined oro-nasopharyngeal swabs were placed into viral transport medium (VTM, containing 26.5 g/L tryptose phosphate broth (Sigma-Aldrich), 5 g/L gelatin (Sigma-Aldrich), 50 mg/l of amphotericin B (Gibco), 100 U/mL Pen/Strep, and 80 mg/l of gentamicin (Gibco), pH 7.2 to 7.4) was taken for molecular SARS-CoV-2 detection by RT-PCR. Swab samples used for virus isolation of Wuhan strains were stored at −80°C for up to 3 months before inoculation of Vero cells and up to 12 months before inoculation of Rhileki cells. Swab samples for virus isolation of α-VoC strains were stored at 4°C for up to 7 days before inoculation of Rhileki and Vero cells. All samples utilized for isolation were obtained as part of first-line testing, analysis, and response of suspected cases through the national outbreak response.

Sera originating from six confirmed COVID-19 patients were identified during surveillance in 2020 and were used to generate a serum pool allowing detection of SARS-CoV-2 infected cells. The antibody titer was determined before by foci reduction neutralization test (FRNT) and sera with FRNT 50 > 100 were pooled. The use of sera was approved by National Ethical Committee for Human Research (No. 206 NECHR). Patient’s information was anonymized prior to the analysis. No primary clinical specimens or identifying information or any other individual-specific information was utilized in these studies or manuscript.

### SARS-CoV-2 detection by RT-PCR.

Molecular detection of SARS-CoV-2 in combined nasopharyngeal/oropharyngeal swabs was similarly performed as previously described (33). RNA extraction was performed using the MagMAX Viral/Pathogen II Nucleic Acid isolation kit (Thermo Fisher Scientific, Waltham, MA, USA), using manual method (200 μL sample input) according to manufacturer’s instructions for the KingFisher instrument (MagMax, Thermo Fisher Scientific). Real-time RT-PCR assays for SARS-CoV-2 RNA detection were performed using primers/probes from Charité Berlin Virology, Germany (34) to detect E gene, and from Pasteur Institute, Paris, France to detect RdRp IP4 gene (35). A 25 μL reaction contained 5 μL of RNA, 12.5 μL of 2 × reaction buffer provided with the Superscript III one step RT-PCR system with Platinum *Taq* Polymerase (Invitrogen, Carlsbad, CA, USA), 0.5 μL of reverse transcriptase/*Taq* mixture from the kit, 0.5 μL of a 50mM magnesium sulfate solution (Invitrogen), and 1 μg of nonacetylated bovine serum albumin (Roche). The qPCR conditions were adjusted as follow: (i) reverse transcription at 55°C for 10 min, (ii) predenaturation at 94°C for 3 min, (iii) 45 cycles of denaturation at 94°C for 15 s and amplification at 58°C for 30 s. The RNA of alpha variant supernatant was detected by real time RT-PCR assay using the PowerChek SARS-CoV-2 S-gene mutation detection version 1.0 kit (Kogene Biotech, Seoul, Korea), according to manufacturer’s instructions (36). The CFX 96 Touch Real-Time PCR (Bio-Rad, Hercules, CA, USA) was used for both RT-PCR assays.

### SARS-CoV-2 detection by foci forming assay.

The detection of infectious virus from virus culture supernatants or staining of SARS-CoV-2-infected cells was performed by FFA, a variation of the viral plaque assay that uses immunostaining to identify infected cells. Supernatant samples were serially diluted in DMEM and then incubated with VeroE6 cells for 1h at 37°C and 5% CO_2_. Afterwards, virus dilutions were replaced by an overlay medium containing 2% carboxymethyl cellulose (CMC; Sigma-Aldrich) in DMEM supplemented with 3% FCS and 100 U/mL Pen-Strep. Infected VeroE6 cells were fixed 16 h to 18 h after inoculation with 4% formaldehyde (General Drugs House Co. Ltd., Bangkok, Thailand) in 1x PBS (Sigma-Aldrich) for 20 min, washed with 1x PBS, then permeabilized with 0.5% Triton X-100 (Sigma-Aldrich) in 1x PBS for 15 min. After an additional washing step, unspecific binding was prevented by incubation with blocking solution (10% FCS in 1xPBS), followed by a 1 h of incubation with a SARS-CoV-2-specific antibody (rabbit; antibodies-online GmbH, Aachen, Germany) targeting the S2 subunit of the viral spike protein and an anti-rabbit IgG HRP conjugated antibody (goat; antibodies-online GmbH). After a washing step, an incubation with an anti-goat IgG horseradish peroxidase conjugated antibody for 1 h, and one more washing step, infected cells were visualized with TrueBlue TMB substrate (KPL). For quantitative evaluation of infected cells or titration of viral supernatants, stained foci were counted with an ELISPOT reader (AID Autoimmune Diagnostika GmbH, Strassberg, Germany).

### Statistical analyses.

Calculations, figures, and statistics were performed using Prism 9.1.2 (GraphPad Software).
