# Supplementary material for: A Look inside the Replication Dynamics of SARS-CoV-2 in Blyth’s Horseshoe Bat (Rhinolophus lepidus) Kidney Cells
Source: Microbiol Spectr. 2022 May 31;10(3):e00449-22. doi: 10.1128/spectrum.00449-22 (PMC9241725; doi:10.1128/spectrum.00449-22)
Supplement: Supplemental file 1 — Tables S1 to S3. Download spectrum.00449-22-s0001.pdf, PDF file, 0.5 MB [file spectrum.00449-22-s0001.pdf]

Supplemental material

Supplementary Table 1: Summary of reports on SARS-CoV-2 culture in cells from bat species other than *Rhinolophus*.

Family	Species	Organ	SARS-CoV-2 Strain	MOI	CPE	Growth	Assay	Reference
Miniopteridae	small bent-winged bat (*Miniopterus pusillus*)	Kidney	HK20	0.1	none	0.10 $\log_{10}$	qRT-PCR	(1)
Molossidae	Mexican/ Brazilian free-tailed bat (*Tadarida brasiliensis*)	Lung	BetaCoV/France/IDF0372/2020	1	N/D	none	FACS	(2)
Pteropodidae	Leschenault's rousette (*Rousettus leschenaultia*)	Intestine	HK20	0.1	none	0.59 $\log_{10}$	qRT-PCR	(1)
		Kidney	HK20	0.1	none	0.15 $\log_{10}$	qRT-pCR	(1)
		Brain	HK20	0.1	none	0.77 $\log_{10}$	qRT-pCR	(1)
		Lung	HK20	0.	none	-0.31$\log_{10}$	qRT-pCR	(1)
		Lung	HKU-001a	0.1	none	none	qRT-PCR	(3)
		Kidney	HKU-001a	0.1	none	none	qRT-PCR	(3)

Family	Species	Organ	SARS-CoV-2 Strain	MOI	CPE	Growth	Assay	Reference
Vespertilionidae	Japanese house bat *(Pipistrellus abramus)*	Kidney	HK20	0.1	Yes	1.71 log_{10}	qRT-PCR	(1)
		Lung	HK20	0.1	none	-0.09 log_{10}	qRT-PCR	(1)
	Lesser bamboo bat *(Tylonycteris pachypus)*	Kidney	HK20	0.1	none	0.82 log_{10}	qRT-PCR	(1)
	Rickett's big-footed bat *(Myotis ricketii)*	Kidney	HK20	0.1	none	0.07 log_{10}	qRT-PCR	(1)
		Lung	HK20	0.1	none	-0.32 log_{10}	qRT-PCR	(1)
	Big Brown bat *(Eptesicus fuscus)*	Kidney (EFK3B)	SARS-CoV-2 USA-WA1/ 2020	>5	No	No	Plaque	(4)
	Natterer's bat *(Myotis nattereri)*	Skin (patagium)	BetaCoV/France/IDF0372/2020	1	N/D	none	FACS	(2)
	Brandt's bat *(Myotis brandtii)*	Skin (patagium)	BetaCoV/France/IDF0372/2020	1	N/D	none	FACS	(2)
	Common serotine bat *(Eptesicus serotinus)*	Brain	BetaCoV/France/IDF0372/2020	1	N/D	none	FACS	(2)
		Kidney	BetaCoV/France/IDF0372/2020	1	N/D	none	FACS	(2)
	Common noctule *(Nyctalus noctule)*	Kidney	BetaCoV/France/IDF0372/2020	1	N/D	none	FACS	(2)
		Liver	BetaCoV/France/IDF0372/2020	1	N/D	none	FACS	(2)

Family	Species	Organ	SARS-CoV-2 Strain	MOI	CPE	Growth	Assay	Reference
Vespertilionidae (continued)	Greater mouse-eared bat *(Myotis myotis)*	Skin (patagium)	BetaCoV/France/IDF0372/2020	1	N/D	none	FACS	(2)
		Brain	BetaCoV/France/IDF0372/2020	1	N/D	none	FACS	(2)
		Nasal epithelium	BetaCoV/France/IDF0372/2020	1	N/D	none	FACS	(2)
		Nerve	BetaCoV/France/IDF0372/2020	1	N/D	none	FACS	(2)
		Macrophage	BetaCoV/France/IDF0372/2020	1	N/D	none	FACS	(2)
		Tonsil	BetaCoV/France/IDF0372/2020	1	N/D	none	FACS	(2)
		Kidney	BetaCoV/France/IDF0372/2020	1	N/D	none	FACS	(2)
		Lung	BetaCoV/France/IDF0372/2020	1	N/D	none	FACS	(2)

Supplementary Table 2: Details on virus isolations for Wuhan strains (n=22)

SARS-CoV-2 isolate	Ct value* of sample#	Ct value* P1 in Vero culture supernatant 6-7dpi	Ct value* P1 in Rhileki culture supernatant 7dpi
**1775**	20.30	19.53	>45
**1775-2**	21.00	19.76	>45
**1821**	29.12	19.00	>45
**1821-3N**	25.28	12.49	>45
**1875**	24.20	12.43	>45
**1876**	22.45	12.91	>45
**2004G**	19.31	12.57	39.46
**2004N**	19.31	13.07	>45
**2018**	22.58	14.01	>45
**2099**	18.75	11.98	>45
**2126**	20.51	12.96	>45
**2265**	18.83	12.20	>45
**2266**	21.41	12.07	>45
**2310**	24.84	12.35	>45
**2690**	23.18	11.55	>45
**2928**	24.10	11.96	>45
**3286**	22.73	11.61	>45
**3473**	25.74	10.65	>45
**3475**	24.00	11.21	>45
**4667**	23.25	10.87	>45
**5401**	19.35	11.13	35.14
**5403**	22.43	10.99	>45

* E gene RT-PCR; # oro-nasopharyngeal swab in VTM

Supplementary Table 3: Details on virus isolations for α-VoC strains (n=75)

SARS-CoV-2 isolate	Ct value* sample[#]	Ct value** p1 in Vero culture supernatant 5-7dpi	Ct value** p1 in Rhileki culture supernatant 7dpi	Ct value** p1 in Rhileki culture supernatant 15dpi	Ct value** p1 in Rhileki cells 15dpi
**12**	14.84	22.4	42.15	>45	19.88
**16**	16.43	17.76	>45	>45	35.76
**18**	16.14	21.96	>45	>45	37.35
**22**	15.13	17.48	30.47	>45	34.23
**23**	22.21	18.06	>45	>45	>45
**25**	16.06	24.73	>45	>45	34.86
**26**	17.75	23.68	>45	35.47	22.18
**27**	19.17	22.02	>45	>45	>45
**28**	24.58	20.28	>45	>45	>45
**29**	17.71	17.25	>45	37.46	>45
**30**	22.19	23.15	>45	>45	>45
**31**	16.41	22.05	>45	43.00	39.02
**33**	16.46	22.01	>45	>45	40.00
**34**	28.05	17.89	>45	>45	42.12
**35**	18.96	20.08	>45	>45	38.62
**40**	20.26	15.07	40.78	41.78	40.38
**41**	20.27	25.32	>45	39.05	33.63
**43**	16.77	28.33	>45	32.72	27.90
**45**	17.46	30.33	>45	30.46	20.34
**46**	17.50	17.67	>45	>45	28.80
**50**	17.03	28.05	>45	>45	34.34
**51**	15.87	26.95	>45	29.97	27.01
**52**	20.12	26.73	>45	>45	35.93
**56**	13.78	28.9	>45	>45	26.58
**57**	18.85	30.34	>45	>45	33.00
**58**	26.48	25.05	>45	>45	44.01
**60**	17.20	25.42	41.33	>45	28.52
**65**	13.60	16.33	>45	42.60	16.59
**69**	16.32	24.01	>45	>45	33.46
**78**	13.88	21.26	>45	38.25	21.23
**79**	17.95	23.77	>45	>45	34.18
**81**	16.45	30.58	>45	>45	32.12
**83**	17.41	22.69	>45	>45	>45

SARS-CoV-2 isolate	Ct value* sample[#]	Ct value** p1 in Vero culture supernatant 5-7dpi	Ct value** p1 in Rhileki culture supernatant 7dpi	Ct value** p1 in Rhileki culture supernatant 15dpi	Ct value** p1 in Rhileki cells 15dpi
**84**	21.21	21.36	>45	>45	>45
**90**	15.14	19.46	>45	33.55	21.10
**102**	14.04	27.87	>45	>45	33.63
**103**	15.64	28.18	>45	23.21	18.25
**106**	17.65	23.68	>45	>45	35.13
**111**	16.71	20.2	37.93	>45	>45
**113**	19.68	20.23	>45	>45	>45
**116**	20.15	22.11	>45	>45	31.94
**124**	18.70	25.67	>45	>45	29.8
**130**	20.48	22.12	>45	>45	>45
**138**	18.41	20.43	>45	>45	>45
**141**	20.30	22.78	>45	>45	31.11
**142**	15.26	23.63	>45	>45	26.41
**182**	17.08	29	>45	30.43	27.36
**183**	18.02	25.14	>45	39.55	29.63
**184**	15.76	30.03	>45	>45	28.56
**186**	17.34	25.16	>45	38.02	28.76
**187**	14.11	33.19	>45	>45	28.85
**195**	24.69	26.66	>45	34.05	31.72
**197**	15.41	22.15	37.00	28.53	22.35
**198**	16.88	27.1	35.84	32.49	22.41
**199**	15.67	22.99	>45	38.19	29.08
**200**	12.00	24.48	33.72	34.00	24.79
**203**	16.02	24.42	>45	34.45	22.56
**206**	22.43	22.77	>45	38.41	>45
**207**	16.43	25.81	>45	31.89	24.69
**208**	17.91	23.14	38.18	36.24	35.24
**215**	16.22	26.91	35.13	29.65	27.98
**217**	19.05	24.23	>45	>45	33.36
**220**	17.53	26.21	>45	>45	32.24
**223**	16.40	25.51	36.79	37.09	29.35
**224**	18.22	30.29	>45	>45	32.29
**226**	16.90	31.69	>45	>45	30.26
**229**	20.37	22.36	>45	>45	35.16
**231**	16.80	30.68	>45	>45	31.27

SARS-CoV-2 isolate	Ct value* sample[#]	Ct value** p1 in Vero culture supernatant 5-7dpi	Ct value** p1 in Rhileki culture supernatant 7dpi	Ct value** p1 in Rhileki culture supernatant 15dpi	Ct value** p1 in Rhileki cells 15dpi
**232**	15.82	25.62	>45	>45	31.10
**234**	17.01	29.32	38.31	38.26	30.60
**237**	20.36	37.48	>45	>45	34.42
**239**	17.53	33.04	>45	>45	30.49
**243**	26.15	32.59	32.68	28.10	28.94
**244**	20.43	25.03	37.86	>45	30.27
**247**	14.52	18.12	34.25	29.05	28.38

*IP4; ** E gene RT-PCR; [#] oro-nasopharyngeal swab in VTM

References

1. Lau SKP, Wong ACP, Luk HKH, Li KSM, Fung J, He Z, Cheng FKK, Chan TTY, Chu S, Aw-Yong KL, Lau TCK, Fung KSC, Woo PCY. 2020. Differential Tropism of SARS-CoV and SARS-CoV-2 in Bat Cells. Emerg Infect Dis 26:2961–2965.

2. Aicher S-M, Streicher F, Chazal M, Planas D, Luo D, Buchrieser J, Nemcova M, Seidlova V, Zukal J, Serra-Cobo J, Pontier D, Schwartz O, Pikula J, Dacheux L, Jouvenet N. 2021. Species-specific molecular barriers to SARS-CoV-2 replication in bat cells. bioRxiv 2021.05.31.446374.

3. Chu H, Chan JF-W, Yuen TT-T, Shuai H, Yuan S, Wang Y, Hu B, Yip CC-Y, Tsang JO-L, Huang X, Chai Y, Yang D, Hou Y, Chik KK-H, Zhang X, Fung AY-F, Tsoi H-W, Cai J-P, Chan W-M, Ip JD, Chu AW-H, Zhou J, Lung DC, Kok K-H, To KK-W, Tsang OT-Y, Chan K-H, Yuen K-Y. 2020. Comparative tropism, replication kinetics, and cell damage profiling of SARS-CoV-2 and SARS-CoV with implications for clinical manifestations, transmissibility, and laboratory studies of COVID-19: an observational study. Lancet Microbe 1:e14–e23.

4. Harcourt J, Tamin A, Lu X, Kamili S, Sakthivel SK, Murray J, Queen K, Tao Y, Paden CR, Zhang J, Li Y, Uehara A, Wang H, Goldsmith C, Bullock HA, Wang L, Whitaker B, Lynch B, Gautam R, Schindewolf C, Lokugamage KG, Scharton D, Plante JA, Mirchandani D, Widen SG, Narayanan K, Makino S, Ksiazek TG, Plante KS, Weaver SC, Lindstrom S, Tong S, Menachery VD, Thornburg NJ. 2020. Severe Acute Respiratory Syndrome Coronavirus 2 from Patient with Coronavirus Disease, United States. Emerg Infect Dis 26:1266–1273.
